# Effects of Moss-Dominated Biocrusts on Soil Microbial Community Structure in an Ionic Rare Earth Tailings Area of Southern China

**DOI:** 10.3390/toxics10120782

**Published:** 2022-12-13

**Authors:** Yongsheng Song, Renlu Liu, Liren Yang, Xiaoyu Xiao, Genhe He

**Affiliations:** 1 Key Laboratory of Agricultural Environmental Pollution Prevention and Control in Red Soil Hilly Region of Jiangxi Province, School of Life Sciences, Jinggangshan University, Ji’an 343009, China; 2 Ji’an Agricultural and Rural Industry Development Service Center, Ji’an 343000, China

**Keywords:** moss-dominated biocrusts, core microbiome, microbial diversity, soil health, rare earth tailings

## Abstract

Moss-dominated biocrusts are widespread in degraded mining ecosystems and play an important role in soil development and ecosystem primary succession. In this work, the soil microbial community structure under moss-dominated biocrusts in ionic rare earth tailings was investigated to reveal the relationship between different types of moss and taxonomy/function of microbiomes. The results showed that microbial community structure was significantly influenced by four moss species (*Claopodium rugulosifolium*, *Orthotrichum courtoisii*, *Polytrichum formosum*, and *Taxiphyllum giraldii*). The microbial assembly was more prominent in *Claopodium rugulosifolium* soil than in the other moss soils, which covers 482 bacterial genera (including 130 specific genera) and 338 fungal genera (including 72 specific genera), and the specific genus is 40% to 1300% higher than that of the other three mosses. Although only 141 and 140 operational taxonomic units (OTUs) rooted in bacterial and fungal clusters, respectively, were shared by all four mosses grown in ionic rare earth tailings, this core microbiome could represent a large fraction (28.2% and 38.7%, respectively) of all sequence reads. The bacterial population and representation are the most abundant, which mainly includes *Sphingomonas*, *Clostridium_sensu_stricto_1*, and unclassified filamentous bacteria and chloroplasts, while the fungi population is relatively singular. The results also show that biocrust dominated by moss has a positive effect on soil microbe activity and soil nutrient conditions. Overall, these findings emphasize the importance of developing moss-dominated biocrusts as hotspots of ecosystem functioning and precious microbial genetic resources in degraded rare-earth mining areas and promoting a better understanding of biocrust ecology in humid climates under global change scenarios.

## 1. Introduction

Biological soil crusts, also known as soil microbial crusts, are organic–inorganic complexes on the uppermost 0–10 mm of the surface soil formed by the bonding of cryptogamous plants such as bacteria, fungi, algae, lichens, and mosses and their hyphae and secretions with soil gravel. They are an important type of surface coverage in arid and semi-arid areas (more than 40%). Biocrusts are the engineer in any soil ecosystem: they can regulate biogeochemical nutrient and water cycles [1,2]; increase soil fertility [3]; stabilize topsoil [4]; and affect the settlement and distribution of soil microorganisms [5], soil animals [6] and vascular plants [7]. Bryophytes are a common pioneer plant in the succession of natural ecosystems, and they are also one of the groups with the largest biomass in biocrusts. Bryophytes are widely distributed and can live in tropical, temperate, and cold areas (such as Antarctica and Greenland) [8]. Bryophytes generally grow in dense mats and have a high capacity for water absorption. They can accumulate water and floating dust in the surrounding environment, secrete acidic metabolites to corrode rocks, and promote the decomposition of rocks to form soil parent material. They also bind soil and hold it together, and are a good indicator of global change and environmental quality [9]. Moss crusts are dominant in the final stage of biocrust development and comprise mainly liverworts and mosses. They are usually clustered in areas such as arid, semi-arid, and cold zones where vascular plants cannot survive or are less widely distributed. Dead bryophytes do not decay and degrade rapidly. With the passage of time, the crust thickens, and its ability to retain water and accumulate nutrients increases. Therefore, the presence of a moss crust greatly improves the soil moisture and physico-chemical properties, and plays an important role in soil genesis, soil quality improvement, and the evolution of vegetation.

Although biocrusts cover about 12% of the land surface in all terrestrial biological communities [10], the studies of these communities at the global level are inconsistent. Most of the foci and research on biocrusts occurs in arid and semi-arid areas, but there is a geographical gap in soil restoration ecology in many other eco-geographical areas, such as degraded rare-earth mining areas [10,11]. Ionic rare-earth ore is a kind of mineral deposit in which rare earth elements are adsorbed on the surface of clay minerals in the ore in the ionic state. Among them, the Gannan rare-earth mining area is a typical ionic rare-earth mining area in China [12]. Traditionally, the heap leaching method is widely used, that is, mining the soil rich in rare earth elements from the mining area, piling it onto isolation mats, and leaching it with ammonium sulfate solution [13]. However, this process has led to the loss of the surface-weathering layer of the mining area, the serious destruction of vegetation, and the exposure of bedrock.

Studies on biocrusts in humid areas are expected to increase due to human disturbance and climate change; in addition, the coverage of biocrusts, especially moss crusts, in these environments is expected to increase. Therefore, moss crust ecology in humid climates (such as the rare earth mining areas in southern China) should be incorporated into the global biocrusts’ scientific research system to enhance a more comprehensive understanding of the functions and services related to biocrusts in terrestrial ecosystems. In addition, improving the understanding of bryophyte crust ecology will contribute to the management and restoration of ecosystems in degraded rare-earth mining areas.

The soil microbial community is an important indicator of ecological restoration of degraded ecosystems [14] and promotes the biogeochemical cycle of the earth [15]. The soil bacterial community is the largest part of soil biodiversity, which exerts a significant influence on ecosystem functions and services [16]. In the past decade, the further understanding of the relationship between environment and microbial ecology has led to many studies focusing on the distribution of soil microbial communities [11], the impact of microbial diversity on plant communities [17], and the versatility of terrestrial ecosystems [10,17]. However, there is limited information on the response of bacterial communities to soil variables in damaged ecosystems. In addition, each microorganism may play different functional roles in complex microbial ecosystems [18]. Experiments suggest that the bacterial communities of the two sample types were similar at the phylum level. The relative abundances of several genera are significantly different at the genus level. However, in most degraded ecosystems, the basic role of bacteria in the key ecosystem process of regulating biocrusts (i.e., nutrient cycling) is unclear; therefore, it is necessary to understand the function of soil microorganisms, especially the relationship between bacteria and fungi and biocrusts’ moss population in a damaged ecosystem.

The early relatively backward pool leaching and heap leaching process in rare earth mining areas in the south of Jiangxi Province (hereafter referred to as “Gannan”) has led to severe damage to the surrounding ecological environment, making it necessary to develop and study techniques and methods for ecological restoration in the Gannan rare earth mining area: in its polluted areas, biocrusts dominated by moss are considered to be one of the most effective means of ecological restoration in the early stages [19,20]. There is a lack of research on moss crusts in the ecosystem of rare earth mining areas in southern China. At present, the relationship between the dominant moss species in moss crusts and their lower soil-microbial groups remains unclear, as is how the dominant moss species affect the underground microbiota. Whether the indigenous dominant mosses in the mining area can be used to improve the soil quality and health of the ecosystem in the rare earth mining area also remains unclear; thus, limiting the process of early ecological restoration in rare earth mining areas in Gannan.

This study aimed to determine the composition and distribution of soil microbial community (bacteria and fungi) diversity in different moss crusts in the ecosystem of the rare-earth tailings area, and ascertain how the composition of the soil microbial community (bacteria and fungi) and soil nutrient content under different moss crusts affect the moss population of biocrusts. A combined method involving field experiments and high-throughput sequencing of the 16S rDNA and ITS gene was used in our study. The results obtained here could provide an important theoretical basis for the restoration of soil ecosystem and the protection of microbial genetic resources in rare earth mining areas in Gannan.

## 2. Materials and Methods

### 2.1. Study Site and Field Sampling

**Figure 1:**
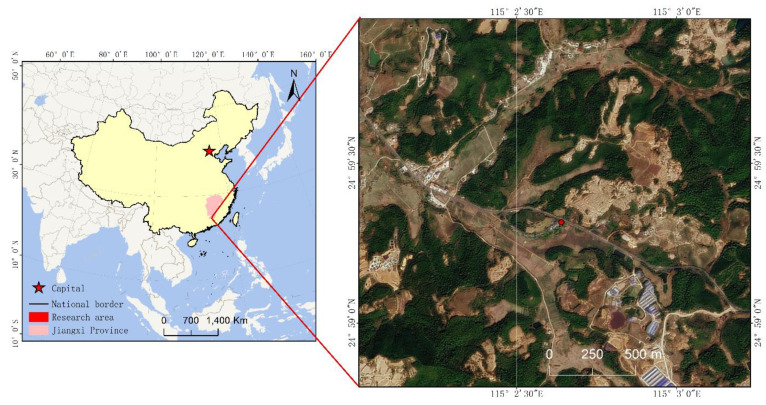
Location of the study site.

Gannan is located on the southern edge of the middle subtropical zone with a humid monsoon climate with sufficient sunshine, an annual average temperature of 18.7 °C and annual rainfall of 1639.8 mm. There are distinct rainy and dry seasons, which directly affect the development of the bryophyte community in the mining area. The moss crust samples were collected near Ganzhou Ruiyuan Biotechnology Co., Ltd. (Ganzhou, China), Dingnan County, Jiangxi Province (115°2′10″ E and 24°59′31″ N) (Figure 1). The company is located in an important pool leaching or heap leaching area for early rare-earth mining. The terrain is mainly mountainous and hilly, with an altitude of 400–500 m. The vegetation in the study area involves mainly artificially planted shrubs and grasses, with few large trees. The moss crusts with large coverage and well-developed species such as *Claopodium rugulosifolium*, *Orthotrichum courtoisii*, *Polytrichum formosum*, and *Taxiphyllum giraldii* were selected. A total of 12 plots were selected in the study area (Figure 1), and each plot was at least 500 m from another plot. Each plot measured 10 m × 10 m. Three pairs of methacrylate tubes (150 mm diameter and 80 mm high) were randomly inserted to a depth of 30 mm in the well-developed moss biocrusts in each plot. The distance between each pair of tubes was at least 5 m. Four biocrust samples were collected at each sampling plot and mixed into one. Soil that loosely adhered to the moss crusts was removed by vigorous shaking, and when no more soil could be removed, the moss crust system was submerged in tubes containing 5 mL of LifeGuard Soil Preservation Solution (Mo Bio Laboratories, Carlsbad, CA, USA) and vigorously shaken to wash the crusts and recover around 1 g of crusts soil per sample for storage in an ice-box and transportation to the laboratory as soon as possible for soil microbial DNA extraction. Other soil samples were used for the analysis of physico-chemical properties and the determination of soil texture and rare earth tailings on pure bare ground were collected as the control to evaluate the basic physico-chemical properties of soil.

### 2.2. Soil Nutrient Analysis

Soil organic matter (OM), total nitrogen (TN), total phosphorus (TP), available nitrogen, and phosphorus were identified as the primary soil nutrients. Soil organic carbon was determined by dichromate oxidation [20]. Total nitrogen was analyzed by the Kjeldahl method [21]. Total phosphorus was determined by the molybdenum blue method [22]. Ammonium nitrogen was studied by Indophenol Blue Colorimetry [23]. Nitrate nitrogen was determined by phenol disulfonic acid colorimetry [21]. Available P was extracted and analyzed by 0.5 M NaHCO_3_ at pH 8.5 [24].

### 2.3. 16 S Amplicon Sequencing and Bioinformatic Processing

Total soil microbial DNA was extracted from 0.5 g freeze-dried soil of each sample using the Fast DNA^®^ Spin Kit for Soil (MP Biomedicals, Santa Ana, CA, USA) in accordance with the manufacturer’s instructions. The final DNA concentration and purification were determined using a NanoDrop 2000 UV–vis spectrophotometer (Thermo Scientific, Wilmington, NC, USA), and the extracted DNA quality was checked by 1% agarose gel electrophoresis. To evaluate the composition and structure of soil bacterial and fungal communities, we amplified the V3–V4 region of the bacterial 16S rRNA gene with primers 338F/806R [25] and the ITS1 region of the fungal ITS rRNA gene with primers ITS1F/ITS2R [26], respectively. The V3–V4 hypervariable regions of bacteria 16SrRNA gene were amplified with primers 338F (5′-ACTCCTACGGGAGGCAGCAG-3′) and 806R (5′-GGACTACHVGGGTWTCTAAT-3′) using a Thermocycler GeneAmp 9700 (Applied Biosystems, South San Francisco, CA, USA). The ITS1 region of fungi was amplified with ITS1_F (5’-CTTGGTCATTTAGAGGAAGTAA-3’) [27] and ITS2 (5′-GCTGCGTTCTTCATCGATGC-3′) [28]. On the Illumina MiSeq platform (Illumina, San Diego, CA, USA) of the Majorbio Pharm Technology Co., Ltd. (Shanghai, China), the sequencing numbers of each sample were rarefied to the sample with the minimum number of reads (3180 reads for 16S rRNA gene and 1682 reads for ITS rRNA gene). The RDP extension to PANDASeq assembler was used to merge the paired-end reads with a minimum overlap of 10 bp and a PHRED score of at least 25 [29,30]. Primer sequences were removed from each sample’s FASTQ file using Flexbar Version 2.5(Revolution Analytics, New York City, NY, USA) [31]. Sequences were converted to FASTA format and concatenated into a single file. All reads were clustered into OTUs using the UPARSE strategy by dereplication, sorting by abundance with at least two sequences and clustering using the UCLUST SmallMem algorithm [32]. These steps were performed with VSEARCH Version 1.0.10 (Revolution Analytics, New York City, NY, USA) [33], which was an open-source, 64-bit multi-threaded-processor-compatible alternative to USEARCH [34]. Chimeric sequences were then detected using the UCHIME algorithm implemented in VSEARCH. All reads before the dereplication step were mapped to OTUs using the usearch_global method implemented in VSEARCH to create an OTU table and converted to BIOM-Format 1.3.1 (Revolution Analytics, New York City, NY, USA) [29]. Finally, taxonomic information for each OTU was added to the BIOM file by using the RDP Classifier Version 2.10 (Revolution Analytics, New York City, NY, USA) [30]. All steps were implemented via the Snakemake workflow [34].

### 2.4. Diversity and Abundance Analysis

For downstream analysis, we took the obtained-OTU table and prepared a “filtered table” using QIIME (1.9.1) custom scripts [35]. First, we extracted from the OTU table the bacteria and fungi domain using the command split_otu_table_by_taxonomy.py. Next, singletons, doubletons, chloroplasts, and mitochondria sequences were discarded using the command filter_otus_table.py. With the “filtered_OTU table,” the alpha diversity was first calculated. Using the command alpha_rarefaction.py, the OTU table was rarefied to counts containing to 35,000 reads. The reason for using this value was because this was the lowest sequencing depth obtained from a sample. To calculate the diversity indices, the alpha_diversity.py and alpha_rarefaction commands were used to determine Shannon, observed OTUs, Chao1, and Faith’s phylogenetic diversity metrics. One-way ANOVA and Tukey HSD, as well as statistical tests to validate ANOVA assumptions, were performed in R (Version 3.4.1) [36]. For the beta diversity, the unrarefied “filtered_OTUtable” was first normalized using the R package metagenomeSeq (Version 1.12, Vienna, Austria) [37]. We used a cumulative-sum scaling (CSS) method to avoid the biases generated with current sequencing technologies due to uneven sequencing depth [38]. With the normalized OTU table Bray–Curtis, weighted and unweighted Unifrac dissimilarity matrices were calculated and used to perform principal coordinate analysis with Phyloseq package (Version 1.10) (Revolution Analytics, New York City, NY, USA) [39]. The non-parametric Adonis test was used to assess the percentage of variation explained by the soil type along with its statistical significance using Vegan (Version 2.4-0) package [36], all performed in R. For differential abundance analysis, STAMP software (Version 2.1.3) (Revolution Analytics, New York City, NY, USA) was used [40]. Rarefied OTU tables from moss biocrusts soil data (35,000 reads per sample) were employed for pairwise comparisons. Welch’s *t*-tests followed by Bonferroni corrections were performed at phylum and at family levels between soils. Dendrograms were established in STAMP with an average neighbor method (UPGMA), and the rows included all the bacterial phyla observed in soil samples along with their relative abundance.

To compare the number of common and proprietary rhizosphere bacterial or fungal genera in different moss crusts, the on-line tool Venny (Version 2.1) (Revolution Analytics, New York City, NY, USA) was used to represent the exclusive genera in a graphical fashion. The Euler diagram was established using the shining application Euler in the R software package with the same name. A Euler graph is a scaled generalized Wien graph, which relaxes the requirements of all intersections. According to the dominant species of bryophytes, Euler diagrams of exclusive and common genera were established.

In order to better understand the composition of bacterial diversity in moss soil, the command Radfit from the R package vegan was used to evaluate several species’ abundance distribution. Species abundance-distribution models were calculated as previously reported [36].

### 2.5. Core Microbiome and Symbiotic Network Analysis

For the core microbiome analyses, rarefied OTU tables (35,000 reads per sample) were used for four soils. The QIIME command compute_core_microbiome.py was applied to obtain a list of OTUs observed in 100% moss crust samples. Core microbiome analyses were conducted for each moss-crust type, in which only the core OTUs with relative abundance >0.5% were represented graphically. Pie and doughnut charts were built in R. Network analysis was performed to assess the complexity of the interaction among microbial taxa in four types of moss crusts (strictly following the best practices of symbiotic network construction) [40]. The sparse OTU table was filtered to a threshold of at least 25 sequences per OTU. SPARCC was used for non-random co-occurrence analysis [35]. The *p*-value was obtained from 99 randomly selected permutations in the data table. The magnitude was >0.8 or ≤0.8, which was deemed statistically significant (*p* < 0.01). The nodes in the reconstructed network represent 97% identity OTU, and the edge corresponds to the strong and significant correlation between nodes. The topology of the network was inferred from a set of metrics (number of nodes and edges, modularity, number of communities, average path length, network diameter, average degree, and clustering coefficient). These metrics were calculated using Gephi (Version 0.9.2) (Revolution Analytics, New York City, NY, USA) [41]. Clusters were constructed using Cytoscape (Version 3.4.0) (Revolution Analytics, New York City, NY, USA) [42,43], hierarchical clustering algorithm (HC-PIN), and Cytoscape plugin CyOMluster [44].

## 3. Results

Using bacterial primers Eub338F/Eub806R, the sample was pre-treated and the quality was filtered to obtain 521,217 sequences, with an average length of 409 bp, roughly covering the amplified V3 region. The low abundance and false OTUs were further filtered to produce 3180 clusters, with a sequence similarity of 97%. The fungal primer ITS1F/AFP308R was used to pre-treat the sample and the quality filtered to obtain 553,626 sequences, with an average length of 246 bp, roughly covering the amplified V3 region. Further filtering low abundance and false OTUs produced 1682 clusters: the sequence similarity was 97%. Some 34,100 sequences were selected as an appropriate depth for sparsity analysis and community uniform sampling.

### 3.1. Physico-Chemical Analysis of Soil under Different Moss-Dominated Biocrusts

**Figure 2:**
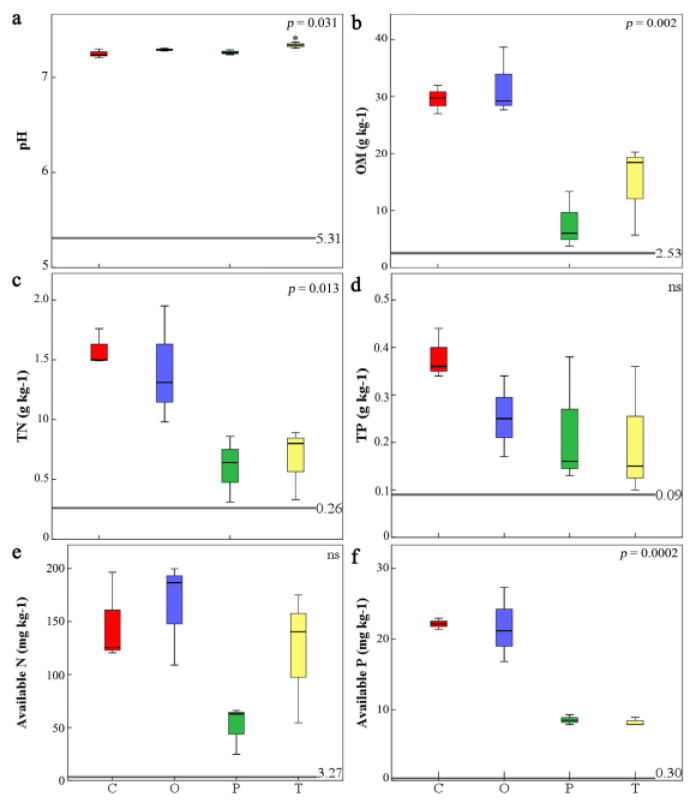
Soil characteristics in pH (**a**), OM (**b**), TN (**c**), TP (**d**), Available N (**e**), and available P (**f**) in four moss-dominated biocrusts and bare soil. The boxplots show the median (center black line) and interquartile ranges (box) of the individual effect sizes. *p* values denote significant differences among magnitudes or duration of manipulation based on Wilcoxon rank-sum test with Bonferroni correction. Horizontal grey solid lines and the number represent the background values of bare soil. OM: Organic matter, TN: Total nitrogen; TP: Total phosphorus; Available N: Available nitrogen, including the sum of ammonium nitrogen and nitrate nitrogen; Available P: Available phosphorus; Red color was assigned to Claopodium rugulosifolium soil; cyan to Orthotrichum courtoisii soil; green to Polytrichum formosum soil; yellow to Taxiphyllum giraldii soil. C: *Claopodium rugulosifolium*; O: *Orthotrichum courtoisii*; P: *Polytrichum formosum*; T: *Taxiphyllum giraldii*; ns: no significant differences. * *p* < 0.05.

Compared with pure bare land, in addition to soil total potassium, the moss-dominated biocrust significantly increases the soil nutrient contents (e.g., soil OM, TN, and TP) and their bioavailability, while also changing the soil pH from acidic to neutral (Figure 2) (Supplementary materials: Table S1). Specifically, compared with bare land, the moss biocrusts significantly increased the amount of soil organic matter, total nitrogen, total phosphorus, available nitrogen, and available phosphorus by 9.18–19.11, 1.31–5.08, 1.22–3.22, 14.80–49.49, and 27.60–72.93 times, respectively.

The biocrusts formed by different bryophyte species exerted different effects on soil nutrients, except for TP (Figure 2d) and available phosphorus (Figure 2e). Among them, *Taxiphyllum giraldii* was significantly stronger than *Claopodium rugulosifolium*, *Orthotrichum courtoisii*, and *Polytrichum formosum* in improving soil acidity (Figure 2a), while *Claopodium rugulosifolium* and *Orthotrichum courtoisii* were significantly higher than those of *Polytrichum formosum* and *Taxiphyllum giraldii* in terms of the amounts present in soil OM (Figure 2b), TN (Figure 2c), and available phosphorus (Figure 2f).

### 3.2. Diversity of Microbial Communities Is Driven by Moss Species

**Figure 3:**
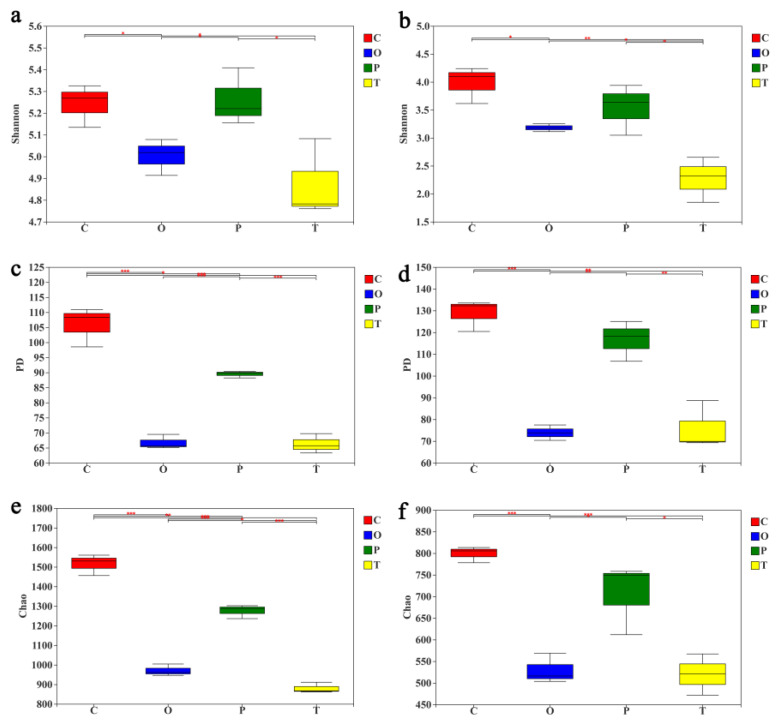
Comparative analysis of the alpha diversity of 16S rRNA and ITS rRNA soil microbe sequences from moss-dominated biocrust soils. (**a**,**b**) Shannon; (**c**,**d**) Phylogenetic diversity; and (**e**,**f**) Chao1 were calculated by moss type. The data were rarefied up to 35,000 counts per sample. The left boxplots show bacteria diversity and the right for fungi. Statistically significant differences (* *p* < 0.05, ** *p* < 0.01, *** *p* < 0.001) were determined by one-way ANOVA followed by post hoc Tukey test. Red color was assigned to *Claopodium rugulosifolium* soil; cyan to *Orthotrichum courtoisii* soil; green to *Polytrichum formosum* soil; yellow to *Taxiphyllum giraldii* soil. C: *Claopodium rugulosifolium*; O: *Orthotrichum courtoisii*; P: *Polytrichum formosum*; T: *Taxiphyllum giraldii*.

The α-diversity, the evenness, represented by the Shannon index and the phylogenetic diversity (PD), were similar in general between bacteria and fungi communities in the *Orthotrichum courtoisii* and *Polytrichum formosum* samples (Figure 3a–d), while the bacterial species richness was significantly higher in the *Orthotrichum courtoisii* soil (Figure 3e). In terms of the diversity of fungal community, the diversity indices (Shannon index, PD index, or Chao index) were generally similar. In general, those of *Clapodium rugulosifolium* were significantly higher than those of *Orthotrichum courtoisii* and *Taxiphyllum giraldii* (Figure 3b,d,f), although there was no significant difference in diversity indices between *Orthotrichum courtoisii* and *Taxiphyllum giraldii* treatments.

**Figure 4:**
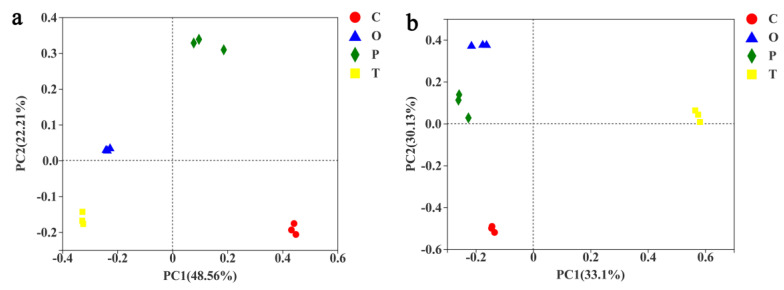
Soil microbial community structure in four moss-dominated biocrusts. Principal Coordinate Analysis (PCoA) of 16S rRNA and ITS rRNA diversity used in this study. (**a**) Soil bacterial community. Moss species explained 48.56% of the total variability in the bacterial community composition (PERMANOVA, *p* < 0.001). (**b**) Soil fungal community. The species of moss determined 33.10% of the total variability in the agricultural soil (PERMANOVA, *p* < 0.001). CSS transformed reads were used to calculate Bray–Curtis distances in (**a**,**b**). Red color was assigned to *Claopodium rugulosifolium* soil and cyan to *Orthotrichum courtoisii* soil and green to *Polytrichum formosum* soil and yellow to *Taxiphyllum giraldii* soil. C: *Claopodium rugulosifolium*; O: *Orthotrichum courtoisii*; P: *Polytrichum formosum*; T: *Taxiphyllum giraldii*.

Regarding β-diversity, Bray–Curtis metrics, and principal component analysis (PCoA) revealed a significant effect of the moss species (Figure 4). PCoA analysis based on Bray–Curtis distance showed that the dominant species of moss significantly affected the microbial community distribution among samples (Permanova, *p* < 0.001). Permanova analysis showed that the dominant species of moss could individually explain 48.56% (Figure 4a) of the overall difference in bacterial community composition and 33.1% (Figure 4b) of that in the fungal community composition.

### 3.3. Specific Differences in Soil Microbial Composition from Different Moss Species

**Figure 5:**
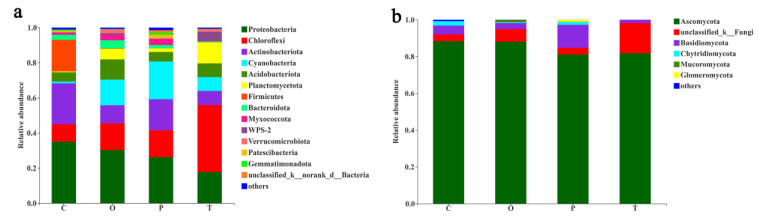
Relative abundance of the most abundant microbial phyla in moss-dominated biocrusts soils. Bar graphs of the relative abundance of the most abundant microbial phyla in the bacterial communities (**a**) and in the fungal communities (**b**) are shown. Only phyla with a total relative abundance higher than 1% are listed separately in the graphs, while less than 1% are reduced to others. C: *Claopodium rugulosifolium*; O: *Orthotrichum courtoisii*; P: *Polytrichum formosum*; T: *Taxiphyllum giraldii*.

The overall diversity of moss-dominated biocrusts was very rich, even in higher classification levels. From the perspective of bacteria, 1240 species that belong to 645 genera, 331 families, 205 orders, 82 classes, and 82 phyla were detected in this study, of which the most abundant bacterial phyla were *Proteobacteria* (27.3%), *Chloroflexi* (19.6%), *Actinobacteriota* (14.8%), *Cynaobacteria* (11.2%), and *Acidobacteriota* (7.5%) (Figure 5a). From the perspective of fungi, 751 species that belong to 495 genera, 254 families, 101 orders, and 14 phyla were detected in this experiment, and the most common phylum was Ascomycota (>81%) (Figure 5b).

Several bacterial populations were affected by different moss species. In terms of bacterial diversity, compared with other moss treatments, *Claopodium rugulosifolium* was more conducive to the emergence of Firmicutes (18.0%), while it was less than 0.2% in other moss-dominated biocrusts (Figure 5a). In *Taxiphyllum giraldii* treatment, *Chloroflexi* (38.2%) was the most abundant phyla, which was significantly higher than that in the other three bryophyte biocrust treatments (Figure 5a). *Acidobacteria* (11.5%) were more abundant in the treatment of *Orthotrichum courtoisii*, which was significantly higher than that in other moss-dominated biocrust treatments (5.2–7.8%) (Figure 5a). In terms of fungal diversity, the abundance of Basidiomycota (12.5%) in the treatment of *Polytrichum formosum* was significantly higher than that of other moss crusts (1.8–4.8%) (Figure 5b), although Ascomycota cover more than 80% of the fungal communities. However, the abundance of an *unclassified_k_fungi* in *Taxiphyllum giraldii* treatment was 16.4%, which was much higher than that in other moss-dominated biocrusts (3.6–6.8%) (Figure 5b). At the genus level, the difference of microbial diversity caused by moss species difference was greater (Figures S1 and S2).

**Figure 6:**
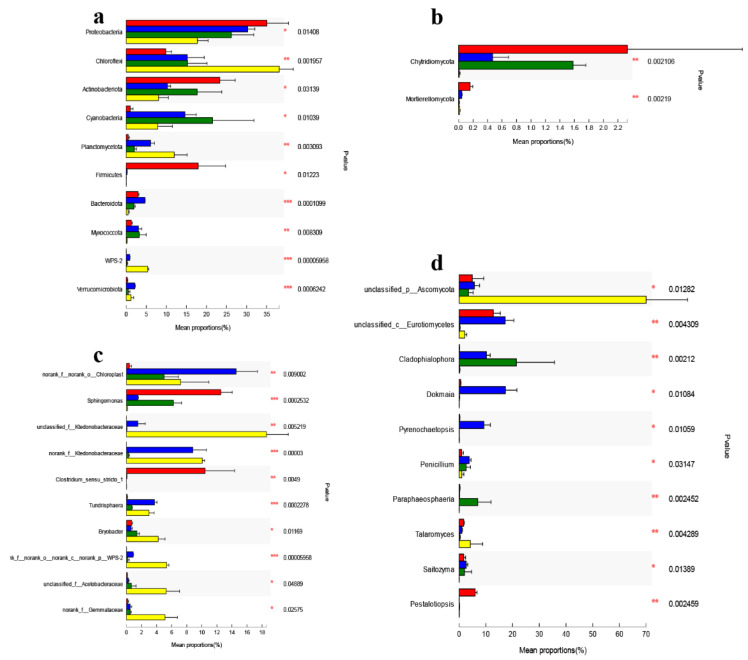
Differential abundance of bacterial or fungal OTUs in moss-dominated biocrusts soils. Welch’s *t*-tests followed by Bonferroni corrections were performed from *Claopodium rugulosifolium*, *Orthotrichum courtoisii*, *Polytrichum formosum*, and *Taxiphyllum giraldii* soil at phylum (**a**,**b**) and genus (**c**,**d**) levels. Only differentially abundant phyla and genus are shown. The left histogram plots show bacteria abundance difference and the right for fungi. Statistically significant differences (* *p* < 0.05, ** *p* < 0.01, *** *p* < 0.001) were determined by one-way ANOVA followed by post hoc Tukey test. Red color was assigned to *Claopodium rugulosifolium* soil; cyan to *Orthotrichum courtoisii* soil; green to *Polytrichum formosum* soil yellow to *Taxiphyllum giraldii* soil.

*Proteobacteria*, *Actinobacteriota*, and *Firmicutes* were consistently more abundant in the soil of *Claopodium rugulosifolium*, while *Chloloflexi*, *Planctomycetota*, and *WPS-2* showed a consistent decrease in soil compared to other moss species (Figure 6a). In contrast to the greater differences in abundances in bacterial populations, only *Chytridiomycota* and *Mortierellomycota* in fungal populations had abundant differences (Figure 6b) (Welch’s *t*-test, *p* < 0.05, Bonferroni-corrected). Among the *Proteobacteria* enriched in *Claopodium rugulosifolium*, *Sphingomonas* was the most abundant genus together with *Clostridium_sensu_stricto_1* for the *Firmicutes* (Figure 6c). The smaller yet significant biocrust effect observed for the fungal communities in the *Claopodium rugulosifolium* soil was elucidated by higher relative abundances of *Pestalotiopsis* and *unclassified_c_Eurotiomycetes* for the *Ascomycota* (Figure 6d).

### 3.4. Higher Diversity of Specific Microorganisms for Claopodium Rugulosifolium in Moss-Dominated Biocrusts

**Figure 7:**
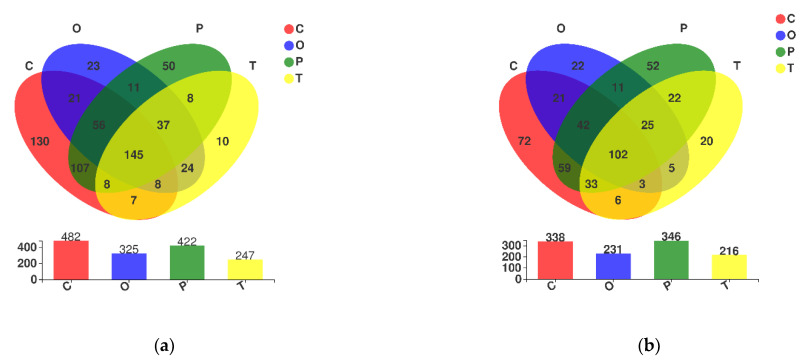
The enriched and depleted microorganism in moss-dominated biocrusts soils. Area-proportional Euler diagrams were built to depict the exclusive and the shared genera. Number of (**a**) bacterial genera and (**b**) fungal genera shared among *Claopodium rugulosifolium*, *Orthotrichum courtoisii*, *Polytrichum formosum*, and *Taxiphyllum giraldii* is depicted within the intersection while the number of genera exclusive to each moss type can be seen out of the intersection zone. The genera exclusive to the *Claopodium rugulosifolium* soil are visible in the red colored area. C: *Claopodium rugulosifolium*; O: *Orthotrichum courtoisii*; P: *Polytrichum formosum*; T: *Taxiphyllum giraldii*.

At the genus level, microbial communities dominated by different moss species were compared to evaluate the impacts of habitat expansion and natural restoration on soil microbial diversity. The results indicated that 130 soil microorganisms in bacteria were specific to *Claopodium rugulosifolium*, accounting for 27.0% of its soil bacterial genera; additionally, these endemic genera accounted for 19.9% (Figure 7a) of the abundance of total bacterial genera for four mosses, which was 2.6–13 times that of other moss types. Although there were only 72 species of endemic fungi in the rhizosphere of *Claopodium rugulosifolium*, accounting for 21.3% of its soil fungal genera, accounting for 14.5% (Figure 7b) of the abundance of total fungal genera for four mosses, the number of endemic genera was 1.4–3.6 times that of other bryophyte groups. In conclusion, more microbial genera (whether bacteria or fungi) in the biocrust soil dominated by *Clapodium rugulosifoliam* were found. It is worth noting that the number and abundance of endemic microbial genera were also the highest.

### 3.5. The Core Microorganisms of Moss-Dominated Biocrusts Are Represented by a Small Subset of Rhizosphere Genera

**Figure 8:**
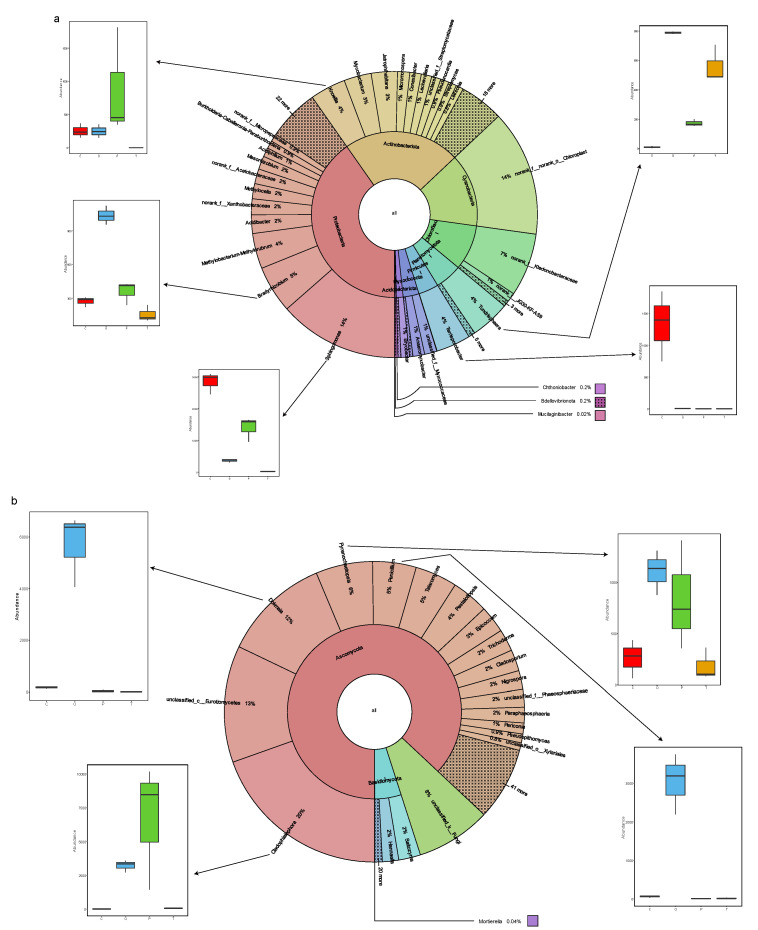
Core microbiome of moss-dominated biocrusts. The different portions within the inner pie chart represent the bacterial (**a**) or fungal (**b**) phyla that are part of the moss core microbiome. The outer donut plot represents the genera that are part of the core, and each genus assigned to the phylum they belong to. The size of the different pie and donut portions represents the contribution of each phylum/genus to the total relative abundance. Satellite box plots depict the relative abundance of selected genera by moss accession (C, O, P, T). Red color was assigned to C; cyan to O; green to P; yellow to T soil, respectively. C: *Claopodium rugulosifolium*; O: *Orthotrichum courtoisii*; P: *Polytrichum formosum*; T: *Taxiphyllum giraldii*.

From the total of 3180 bacterial clustered OTUs, 141 OTUs were found to be consistently present in the rhizosphere soil of all four bryophyte crusts (Supplementary materials: Table S2). These 141 OTUs were classified into genus level, accounting for only 4.43% of the total number of OTUs and 28.2% of all sequences. This core moss rhizosphere bacterial population consists of 54 *Proteobacteria* OTUs, accounting for 40.3% of the average relative abundance. *Sphingomonas* makes the highest contribution (5 OTUs), followed by *Bradyrhizobium* (2 OTUs), and *Methylbacterium methylrubrum* (3 OTUs) (Figure 8a). Other phyla represented in the core rhizosphere microbiota are *Actinobacterita* (37 OTUs, relative abundance 22.7%), *Cyanobacteria* (11 OTUs, 14.2%), *Chloroflexi* (6 OTUs, 8.8%), *Plantomycetota* (11 OTUs, 5.2%), *Firmicutes* (2 OTUs, 3.9%), *Myxococcota* (7 OTUs, 3.1%), *Acidobacterita* (6 OTUs, 1.3%), *Verrucomicrobiota* (1 OTU, 0.2%), *Bdellovibrionota* (5 OTUs, 0.2%), and *Bacteroidota* (1 OTU, 0.1%).

Through analyzing the fungal communities in the subcutaneous soil of these four bryophytes, it was found that 140 OTUs (Supplementary materials: Table S3) of 1682 aggregated fungal groups were always present in each biocrust soil. These 140 OTUs were classified into genus level, accounting for only 8.32% of the total OTUs and 38.7% of the total sequences. This core bryophyte rhizosphere fungus group was composed of 101 *Ascomycota* OTUs, accounting for 87% of the average relative abundance. The contribution of *Cladophialophora* was the highest (2 OTUs), followed by *unclassified_c_Eurotiomycetes* (4 OTUs), *Dokmaia* (1 OTU), and *Pyrenochaetopsis* (2 OTUs) (Figure 8b). The other phyla represented in the core rhizosphere fungi were *Basidiomycota* (32 OTUs, relative abundance 4.9%) and *unclassified_k__Fungi* (5 OTUs, 8.06%) and *Mortierellomycota* (2 OTUs, 0.04%).

In conclusion, these comparative analyses showed that only a few of the 141 bacterial OTUs and 140 fungal OTUs were omnipresent in the four types of moss-crust rhizosphere soil, and showed that these OTUs accounted for more than one quarter and one third of the total number of sequence-reads (28.2% and 38.7%).

### 3.6. Effects of Core Microorganisms on s

**Figure 9:**
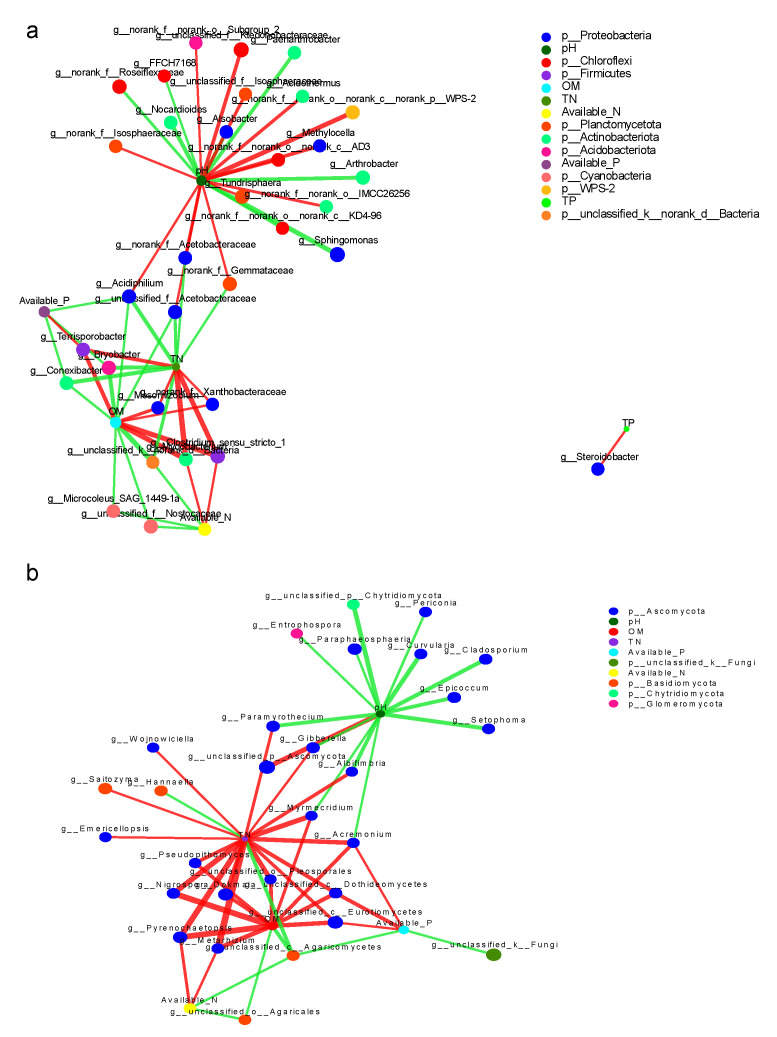
Microorganism and soil characteristics co-occurrence networks in moss-dominated biocrusts soils. (**a**) Co-occurrence network of bacteria. (**b**) Co-occurrence network of fungi. Positive interactions are depicted as red edges and the negative interactions are depicted as green edges.

Two-factor correlation network analysis was used to evaluate the influence of soil core microorganisms in moss crust on soil properties. From the perspective of bacterial flora, pH was co-regulated by 5 bacterial phyla and 22 bacterial genera, of which 14 bacterial genera played a positive correlation regulation (*g_norank_p_WPS-2* had the greatest influence) and 8 bacterial genera had a negative correlation (*g_sphingomonas _p_Proteobacteria* had the greatest impact) (Figure 9a). OM was jointly regulated by 6 bacterial phyla and 12 bacterial genera, of which 5 were positively regulated and 7 were negatively regulated. TN was jointly regulated by 8 bacterial phyla and 11 bacterial genera, of which 5 were positively regulated and 6 were negatively regulated. Despite exerting little influence, available N and available P were also affected by four or five bacterial genera, respectively.

From the perspective of fungi, pH could regulated 14 fungal genera of 2 fungi phyla, of which only *g_unclassified_p_ascomycetes* is a positive correlation, the remaining 13 were negatively correlated (therein *g_unclassified_p_chytridiomycota* had the greatest impact) (Figure 9b). OM was regulated by 12 fungal genera of two phyla of bacteria, including 10 fungal genera with a positive connection and 2 bacterial genera with a negative correlation. TN was regulated by 18 fungal genera of 2 phyla of fungi, including 16 fungal genera with a positive connection and 2 fungal genera with a negative correlation. Akin to the influence of bacterial flora, available N and available P were also affected by four fungal genera, respectively, although the influence thereof was small.

In conclusion, TN, OM, and pH in soil parameters are affected by microbial population, while TP, available N, and available P are less vulnerable to, or weakly affected by, microorganisms. Our goal is to use the betweenness centrality (BC) to determine the core microorganisms in the early moss-crusting stage of ecological restoration of the ionic rare-earth tailings pile and to find key species in this stage. According to the results of this experiment, the highest BC values of bacterial flora were found for the genera *Sphingomonas* (OTU_63), *g_norank_f_ktedonobacterae* (OTU_1215), *g_norank_f_norank_o_ chloroplast* (OTU_1464 and OTU_2886), *Bradyrhizobium* (OTU_2481), *knoellia* (OTU_2534), and *Terrisporobacter* (OTU_660). For the fungal communities, the highest BC values were found for *Terrisporobacter* (OTU_680, phylum), *Dokmaia* (OTU 800), and unclassified *Ascomycota* (OTU_616), unclassified_fungi (OTU_235), and *Pyrenochaetopsis* (OTU_836).

## 4. Discussion

In this study, the microbial diversity of biocrust soil dominated by Claopodium rugulosifolium was found to be higher than that of the other bryophyte species. Through field observations, it was found that bryophytes are widely distributed on the surface of the soil in the mining area, and even on some severely polluted slopes and rocks, indicating that bryophytes are more adaptable to the wasteland in rare earth mining areas and can be used as preferred indicators for mining-wasteland ecological restoration. Although bryophytes are widely distributed in other good habitats [16,45], their distribution in abandoned mines with poor habitats has been less reported. In addition, species’ abundance analysis reveals the niche process of the crust of the four mosses. This shows that selection pressure and soil factors, especially low pH value, are the most likely selection pressure on bare land. Bacterial diversity in acid soil is usually low [41,45]; thus, the pH value largely determines the basic composition of the soil microbial community [46]. Our results further show that the dominant species of moss also have a significant impact on the composition of rhizosphere microbial community, especially in *Cloopodium rugulosifolium*. In the dominant biocrust soil, the rhizosphere effect is greater, and the impact on the diversity composition of rhizosphere microbial community is greater. Its influence presents the following aspects: *Clapodium rugulosifolium* > *Polytrichum formosum* > *Orthotrichum courtoisii* > *Taxiphyllum giraldii*. A potential mechanism of this larger and more uniform rhizosphere effect is that poor abiotic conditions in the soil may affect the quantity and quality of root exudates released by bryophytes into the soil. In the native soil used in this study, bryophytes face low soil pH, low nitrogen, low organic matter, and low phosphorus availability, which is a common problem in the soil of mine tailings [47], and Sphagnum rugosa can better adapt to these harsh habitats.

In terms of bacterial and fungal groups, the biocrust soils dominated by *Clapodium rugulosifolium* has more specific OTUs than the other bryophyte biocrust soils. The specific microflora in biocrust soil dominated by *Clapodium rugulosifolium* reach 130 genera (much higher than that of the other three bryophytes (10–50 genera, Figure 7a)); this feature is also reflected in the fungal community (Figure 7b). The most typical bacterial genera in the biocrusts of *Claopodium rugulosifolium* are *Nitrosomonas* and *Chroococcidiopsis_pcc_7203*, and the fungal genera are *Laccaria* and *Karstenia*. Their abundance is strongly regulated by soil types and negatively affected by low pH [48].

In the biocrust soil dominated by these four mosses, the common core microbiome members are usually very rich. The core bacterial flora includes *g_norank_f_norank_o_chloroplast*, *Sphingomonas*, *Bradyrhizobium*, and *Ktedonobacterae*. These results show that a considerable part of the core bacterial flora of mosses has nitrogen fixation ability and a part has toxin degradation ability [49,50,51,52]. This is an important feature of microorganisms in the early succession of the tailings ecosystem. However, for other non-bryophyte species, these rhizosphere bacteria are not necessarily members of the core rhizosphere microbiota [53]. Among the fungal flora, the core flora include *Cladophialophora*, *Dokmaia*, and *Eurotiomycetes*, which have obvious advantages in inhibiting bacteria, secreting amylase and oxidase, promoting protein, transforming starch into monosaccharides, and catalyzing the oxidation of polyphenols [54,55]. These microbial genera are likely to better adapt to acidic and barren conditions in soil and may respond more effectively to moss root signals, such as flavonoids, benzenes, and monoterpenes released by mosses.

The co-occurrence network analysis further showed that the microbial groups (bacteria and fungi) in the soil of moss crusts growing in the soil environment of rare earth mining wasteland. The interaction between fungi is simpler than that in natural habitat soil, and it is advantageous to establish a co-trophic body in a moss rhizosphere chamber. On the other hand, the common network of fungal flora is also simpler than that of bacterial flora, which may be because fungi have a mycelial network [53]; thus, compared with bacteria, they show stronger resistance and better adaptability to adverse environmental interference. In the microbial common network for bryophyte crust soil, the positive interaction between nodes was observed, indicating niche overlap, and negative interaction was found, indicating competition or alkalization [40,46]. Univariate correlations found that the occurrence of phylogenetically related OTU is usually positively correlated. The relevant literature has shown that the probability of common occurrence of *Acidobacteria* and *Bacillus verrucosa* is higher than expected [56]. In this study, a similar pattern in bryophyte crust soil network was determined. In addition, clustering shows strong niche differentiation [46,57], whether these hubs represent different functional groups remains to be studied through metagenomics and trait-based bioassay.

Plant domestication and subsequent improvement of crop varieties have led to phenotypic, genomic, and metabolic changes that enable humans to make use of plants [19,58]. Many of these changes are accompanied by other inadvertent effects, such as the reduction in the genetic diversity of domesticated plant varieties [59], and the negative impact of domesticated plants on herbivorous insects [60]. Regarding the impact of plant domestication on the rhizosphere microbial community, differences in microbial community structure related to wild and cultivated plant species have been found repeatedly. Similarly, a decrease or increase in the abundance of some taxa in domesticated and wild plants has been observed. The research conducted shows that compared with the domesticated similar plants with sugar beet [61] and barley [62], the rhizosphere Bacteroides of wild-related plants are rich in members [63]. This special enrichment of Bacteroidetes in the bryophyte rhizosphere is not observed in local soil, which may be related to the poor abiotic conditions of the rare-earth tailings matrix. Although there are significant differences in the abundance of microbial groups in biocrust soil dominated by different bryophytes, the mechanism, genotype, and phenotypic characteristics or chemical interactions behind these changes and their effects on other plants, further research is warranted to determine which factors impact the health and development of soil ecology. The results of this study showed that the biological crust dominated by moss significantly improved soil fertility and soil microbial diversity, although the soil microbial groups induced by the different dominant moss species were significantly different. In conclusion, the research results of this paper can provide basic support for promoting the soil improvement process of abandoned mine land by using target moss and accelerating the ecological restoration practice of Ion-type rare earth mining area.

## 5. Conclusions

In this study, the microbial diversity and structural characteristics of different bryophyte crusts in the ecosystem of the tailings yards in ionic rare-earth mining areas in southern China were studied. The results showed that the formation of bryophyte crusts can significantly improve soil nutrient conditions and promote the healthy development of soil ecology. In addition, the role of bryophyte crusts formed by different bryophyte species in improving soil ecology was explored. There are significant differences in the functions and services of the system. In the rare-earth mining area of Southern Jiangxi, the moss biocrusts dominated by *Clopodium rugulosifolium* significantly improved the correlation among soil bacterial abundance, diversity, and species, which was also reflected in the fungal flora. Bryophyte biological crusts, dominated by *Orthotrichum courtoisii* and *Taxiphyllum giraldii*, can greatly improve the soil ecosystem function, but their influences are much weaker than that of *Claopodium rugulosifolium*. Therefore, for the early ecological restoration of rare-earth mining areas in southern Jiangxi, moss may be a pioneer species that can be given priority. In general, our research results show that it is important to cultivate targeted moss-crust materials and develop effective methods to promote the ecological restoration of tailings yards in ionic rare-earth mining areas in southern China. These can not only improve the restoration of ecosystem functions related to moss biocrusts and reduce the risk of soil degradation and soil erosion, but also preserve them as valuable microbial genetic resources. These seed resources are of greater significance to ionic rare-earth mining areas and the ecological restoration of other types of mining wasteland has important biotechnology value.

## Data Availability

The data presented in this study are available on request from the corresponding author. The data are not publicly available due to privacy restrictions.

## Supplementary Materials

The following supporting information can be downloaded at: https://www.mdpi.com/article/10.3390/toxics10120782/s1, Table S1: Partial soil characterization of biological soil crusts dominated by moss and bare soil; Table S2: Total number of core bacterial sequences in four moss-dominated biocrusts; Table S3: Total number of core fungal sequences in four moss-dominated biocrusts.
