# Supplementary material for: Effects of Moss-Dominated Biocrusts on Soil Microbial Community Structure in an Ionic Rare Earth Tailings Area of Southern China
Source: Toxics. 2022 Dec 13;10(12):782. doi: 10.3390/toxics10120782 (PMC9781051; doi:10.3390/toxics10120782)
Supplement: Supplementary file 1 [file toxics-10-00782-s001.zip › toxics-2066425-supplementary.pdf]

toxics

Supplementary Materials: Effects of Moss-Dominated Biocrusts on Soil Microbial Community Structure in an Ionic Rare Earth Tailings Area of Southern China

Yongsheng Song, Renlu Liu, Liren Yang, Xiaoyu Xiao and Genhe He

Table S1. Partial soil characterization of biological soil crusts dominated by moss and bare soil.

Item	Biological soil crusts dominated by moss				Bare soil
	C	O	P	T	
pH	7.25 ± 0.03	7.29 ± 0.01	7.26 ± 0.01	7.34 ± 0.17	5.31 ± 0.01
Total N (g kg^{-1})	1.58 ± 0.09	1.41 ± 0.28	0.60 ± 0.16	0.67 ± 0.17	0.26 ± 0.12
Total P (g kg^{-1})	0.38 ± 0.08	0.25 ± 0.11	0.22 ± 0.15	0.20 ± 0.15	0.09 ± 0.01
Total K (g kg^{-1})	39.12 ± 0.50	39.71 ± 1.56	17.71 ± 5.74	40.61 ± 1.68	44.99 ± 1.17
OM (g kg^{-1})	29.57 ± 1.43	31.82 ± 3.43	7.72 ± 2.88	14.79 ± 4.57	2.53 ± 0.32
Available N (mg kg^{-1})	147.48 ± 24.52	165.10 ± 28.32	51.18 ± 13.24	123.27 ± 35.82	3.27 ± 1.35
Available P (mg kg^{-1})	22.18 ± 0.45	21.78 ± 3.04	8.58 ± 0.40	20.26 ± 0.34	0.30 ± 0.15

C: *Claopodium rugulosifolium*, O: *Orthotrichum courtoisii*, P: *Polytrichum formosum*, T: *Taxiphyllum giraldii.*

Table S2. Total number of core bacterial sequences in four moss-dominated biocrusts.

OTU ID	Number of sequences				Taxonomy		
	C	O	P	T	Genus	Family	Phylum
OTU1720	1	172	14	19	Chthoniobacter	Chthoniobacteraceae	Verrucomicrobiota
OTU2414	2	1603	265	295	Acidibacter	unclassified_o__Gammaproteobacteria_Incertae_Sedis	Proteobacteria
OTU2810	1	73	180	804	Acidiphilium	Acetobacteraceae	Proteobacteria
OTU2778	61	21	16	21	alphaI_cluster	Beijerinckiaceae	Proteobacteria
OTU2481	806	2824	943	449	Bradyrhizobium	Xanthobacteraceae	Proteobacteria
OTU386	12	288	134	22	Bradyrhizobium	Xanthobacteraceae	Proteobacteria
OTU2984	1	568	63	275	Burkholderia-Caballeronia-Paraburkholderia	Burkholderiaceae	Proteobacteria
OTU1423	379	117	133	2	Devosia	Devosiaceae	Proteobacteria
OTU2174	114	65	19	1	Devosia	Devosiaceae	Proteobacteria
OTU2638	64	2	181	1	Ellin6067	Nitrosomonadaceae	Proteobacteria
OTU1820	6	4	58	1	Enterobacter	Enterobacteriaceae	Proteobacteria
OTU2215	62	6	83	17	Hyphomicrobium	Hyphomicrobiaceae	Proteobacteria
OTU1843	5	15	33	2	IS-44	Nitrosomonadaceae	Proteobacteria
OTU1684	1	24	1	8	Legionella	Legionellaceae	Proteobacteria
OTU563	42	17	57	2	Massilia	Oxalobacteraceae	Proteobacteria
OTU618	82	9	69	3	Massilia	Oxalobacteraceae	Proteobacteria
OTU764	25	4	11	1	Massilia	Oxalobacteraceae	Proteobacteria
OTU1622	153	1257	129	2	Mesorhizobium	Rhizobiaceae	Proteobacteria
OTU1102	46	114	377	9	Methylobacterium-Methylorubrum	Beijerinckiaceae	Proteobacteria
OTU1338	67	1769	1273	167	Methylobacterium-Methylorubrum	Beijerinckiaceae	Proteobacteria
OTU466	46	46	178	7	Methylobacterium-Methylorubrum	Beijerinckiaceae	Proteobacteria
OTU3120	11	637	117	990	Methylocella	Beijerinckiaceae	Proteobacteria
OTU2785	75	42	156	39	Methylorosula	Beijerinckiaceae	Proteobacteria
OTU1695	22	11	5	1	MND1	Nitrosomonadaceae	Proteobacteria
OTU1565	1	588	394	583	norank	Acetobacteraceae	Proteobacteria
OTU2732	1	17	57	80	norank	Acetobacteraceae	Proteobacteria
OTU1241	5	18	39	7	norank	Caulobacteraceae	Proteobacteria
OTU2234	1	8	652	30	norank	Caulobacteraceae	Proteobacteria
OTU2662	2	3	7	2	norank	Holosporaceae	Proteobacteria
OTU1337	18	600	246	40	norank	Micropepsaceae	Proteobacteria
OTU1230	5	135	113	34	norank	Mitochondria	Proteobacteria
OTU1348	1	63	32	1	norank	Rhodospirillaceae	Proteobacteria
OTU1221	193	364	35	121	norank	Xanthobacteraceae	Proteobacteria

OTU1535	87	120	204	4	norank	Xanthobacteraceae	Proteobacteria
OTU2688	125	73	54	52	norank	Xanthobacteraceae	Proteobacteria
OTU867	250	44	101	11	norank	Xanthobacteraceae	Proteobacteria
OTU2208	16	15	27	2	Novosphingobium	Sphingomonadaceae	Proteobacteria
OTU831	119	5	20	1	Novosphingobium	Sphingomonadaceae	Proteobacteria
OTU1067	297	9	127	1	Phenylobacterium	Caulobacteraceae	Proteobacteria
OTU1286	4	16	134	1	Phenylobacterium	Caulobacteraceae	Proteobacteria
OTU1350	34	374	128	6	Reyranella	Reyranellaceae	Proteobacteria
OTU472	16	1	1	1	Reyranella	Reyranellaceae	Proteobacteria
OTU1821	47	39	161	1	Rhizobacter	Comamonadaceae	Proteobacteria
OTU1202	61	98	90	4	Rhodoplanes	Xanthobacteraceae	Proteobacteria
OTU866	119	4	1	6	Rhodoplanes	Xanthobacteraceae	Proteobacteria
OTU1026	42	1	3	2	Sphingoaurantiacus	Sphingomonadaceae	Proteobacteria
OTU1389	32	5	29	4	Sphingomonas	Sphingomonadaceae	Proteobacteria
OTU2026	829	283	1088	12	Sphingomonas	Sphingomonadaceae	Proteobacteria
OTU63	6147	633	2209	18	Sphingomonas	Sphingomonadaceae	Proteobacteria
OTU735	142	83	43	2	Sphingomonas	Sphingomonadaceae	Proteobacteria
OTU892	1377	99	821	37	Sphingomonas	Sphingomonadaceae	Proteobacteria
OTU2024	1	47	81	50	unclassified	Acetobacteraceae	Proteobacteria
OTU1620	31	20	15	1	unclassified	Comamonadaceae	Proteobacteria
OTU1623	76	157	28	1	unclassified	Comamonadaceae	Proteobacteria
OTU1947	59	7	17	2	unclassified	Comamonadaceae	Proteobacteria
OTU3015	15	11	7	19	Aquisphaera	Isosphaeraceae	Planctomycetota
OTU2802	2	1	1	3	norank	Gemmataceae	Planctomycetota
OTU1613	3	89	9	13	norank	Isosphaeraceae	Planctomycetota
OTU2389	1	1	16	76	norank	Isosphaeraceae	Planctomycetota
OTU3075	1	51	1	151	norank	Isosphaeraceae	Planctomycetota
OTU1212	1	25	66	5	Singulisphaera	Isosphaeraceae	Planctomycetota
OTU1331	3	464	6	4	Tundrisphaera	Isosphaeraceae	Planctomycetota
OTU1402	3	1170	188	293	Tundrisphaera	Isosphaeraceae	Planctomycetota
OTU1970	20	697	279	1180	Tundrisphaera	Isosphaeraceae	Planctomycetota
OTU2345	5	36	50	206	Tundrisphaera	Isosphaeraceae	Planctomycetota
OTU3171	1	1	2	205	unclassified	Isosphaeraceae	Planctomycetota
OTU1290	3	1306	35	4	Anaeromyxobacter	Anaeromyxobacteraceae	Myxococcota
OTU1372	9	72	43	5	Haliangium	Haliangiaceae	Myxococcota
OTU739	43	1	1	2	Haliangium	Haliangiaceae	Myxococcota
OTU1409	1	23	2	1	Pajaroellobacter	Polyangiaceae	Myxococcota
OTU3069	1	11	6	2	Pajaroellobacter	Polyangiaceae	Myxococcota
OTU1786	5	4	1418	2	unclassified	Myxococcaceae	Myxococcota
OTU2352	1	96	83	1	unclassified	Polyangiaceae	Myxococcota
OTU660	3985	13	1	3	Terrisporobacter	Peptostreptococcaceae	Firmicutes
OTU1099	36	1	1	1	unclassified	Lachnospiraceae	Firmicutes
OTU1460	19	13	1	1	norank	norank_o__Chloroplast	Cyanobacteria

OTU1464	52	5744	859	531	norank	norank_o__Chloroplast	Cyanobacteria
OTU1662	7	416	16	1	norank	norank_o__Chloroplast	Cyanobacteria
OTU175	30	29	98	68	norank	norank_o__Chloroplast	Cyanobacteria
OTU1772	41	401	172	1	norank	norank_o__Chloroplast	Cyanobacteria
OTU1818	15	15	67	4	norank	norank_o__Chloroplast	Cyanobacteria
OTU2264	3	314	55	1	norank	norank_o__Chloroplast	Cyanobacteria
OTU2886	108	1432	367	2432	norank	norank_o__Chloroplast	Cyanobacteria
OTU2888	4	11	6	258	norank	norank_o__Chloroplast	Cyanobacteria
OTU2891	3	94	910	11	norank	norank_o__Chloroplast	Cyanobacteria
OTU1664	2	3	1	1	norank	Obscuribacteraceae	Cyanobacteria
OTU1589	1	988	3	1	norank	JG30-KF-AS9	Chloroflexi
OTU2258	1	10	41	12	norank	JG30-KF-AS9	Chloroflexi
OTU1215	2	7120	20	174	norank	Ktedonobacteraceae	Chloroflexi
OTU2261	2	4	41	107	norank	norank_o__norank_c__AD3	Chloroflexi
OTU2730	2	16	75	66	norank	fnorank_o__norank_c__TK10	Chloroflexi
OTU3112	1	20	9	339	norank	norank_o__norank_c__TK10	Chloroflexi
OTU1209	22	18	16	1	Bdellovibrio	Bdellovibrionaceae	Bdellovibrionota
OTU1320	1	42	17	1	Bdellovibrio	Bdellovibrionaceae	Bdellovibrionota
OTU1980	2	2	14	4	Bdellovibrio	Bdellovibrionaceae	Bdellovibrionota
OTU1185	17	1	5	1	norank	norank_o__0319-6G20	Bdellovibrionota
OTU1410	3	2	5	1	norank	norank_o__0319-6G20	Bdellovibrionota
OTU2151	2	7	15	1	Mucilaginibacter	Sphingobacteriaceae	Bacteroidota
OTU1480	4	22	1	4	Acidothermus	Acidothermaceae	Actinobacteriota
OTU2812	4	78	3	521	Acidothermus	Acidothermaceae	Actinobacteriota
OTU2729	93	173	78	108	Actinomycetospora	Pseudonocardiaceae	Actinobacteriota
OTU152	249	20	67	2	Actinoplanes	Micromonosporaceae	Actinobacteriota
OTU1755	18	41	91	2	Amycolatopsis	Pseudonocardiaceae	Actinobacteriota
OTU1439	180	11	80	1	Blastococcus	Geodermatophilaceae	Actinobacteriota
OTU1641	1	270	94	681	Conexibacter	Solirubrobacteraceae	Actinobacteriota
OTU2991	1	4	4	274	Conexibacter	Solirubrobacteraceae	Actinobacteriota
OTU1471	7	4	6	1	Friedmanniella	Propionibacteriaceae	Actinobacteriota
OTU1432	7	1022	373	117	Jatrophihabitans	Frankiaceae	Actinobacteriota
OTU1433	9	551	430	75	Jatrophihabitans	Frankiaceae	Actinobacteriota
OTU1443	4	258	178	15	Jatrophihabitans	Frankiaceae	Actinobacteriota
OTU1444	19	17	2	1	Kibdelosporangium	Pseudonocardiaceae	Actinobacteriota
OTU2534	760	745	2622	2	Knoellia	Intrasporangiaceae	Actinobacteriota
OTU170	1177	25	16	5	Lechevalieria	Pseudonocardiaceae	Actinobacteriota
OTU1437	77	226	52	7	Leifsonia	Microbacteriaceae	Actinobacteriota
OTU2419	96	47	352	4	Leifsonia	Microbacteriaceae	Actinobacteriota
OTU283	1151	166	93	3	Micromonospora	Micromonosporaceae	Actinobacteriota
OTU123	79	8	13	4	Mycobacterium	Mycobacteriaceae	Actinobacteriota
OTU163	2610	207	87	160	Mycobacterium	Mycobacteriaceae	Actinobacteriota
OTU1541	1	19	14	3	Nocardia	Nocardiaceae	Actinobacteriota

OTU2706	23	1	7	2	norank	Microtrichaceae	Actinobacteriota
OTU1999	129	160	264	1	norank	norank_o__Frankiales	Actinobacteriota
OTU2436	9	11	131	1	norank	norank_o__Frankiales	Actinobacteriota
OTU2955	1	2	3	788	norank	norank_o__IMCC26256	Actinobacteriota
OTU2652	3	1	20	5	norank	norank_o__Microtrichales	Actinobacteriota
OTU2986	1	6	17	273	norank	Solirubrobacteraceae	Actinobacteriota
OTU1354	38	242	86	59	Pseudonocardia	Pseudonocardiaceae	Actinobacteriota
OTU1979	36	1	344	1	Pseudonocardia	Pseudonocardiaceae	Actinobacteriota
OTU535	141	6	1	16	Pseudonocardia	Pseudonocardiaceae	Actinobacteriota
OTU1430	16	15	8	7	Rhodococcus	Nocardiaceae	Actinobacteriota
OTU639	80	23	2	16	Solirubrobacter	Solirubrobacteraceae	Actinobacteriota
OTU158	616	48	282	6	Streptomyces	Streptomycetaceae	Actinobacteriota
OTU1390	27	110	285	26	unclassified	Frankiaceae	Actinobacteriota
OTU2420	101	41	552	2	unclassified	Kineosporiaceae	Actinobacteriota
OTU2061	56	580	337	98	unclassified	Streptomycetaceae	Actinobacteriota
OTU2994	1	2	1	3	unclassified	unclassified_o__Solirubrobacterales	Actinobacteriota
OTU2627	12	128	259	5	Bryobacter	Bryobacteraceae	Acidobacteriota
OTU2693	4	47	54	467	Bryobacter	Bryobacteraceae	Acidobacteriota
OTU1554	3	54	12	1	Candidatus_Solibacter	Solibacteraceae	Acidobacteriota
OTU1678	2	18	25	6	Candidatus_Solibacter	Solibacteraceae	Acidobacteriota
OTU3117	8	15	16	125	Candidatus_Solibacter	Solibacteraceae	Acidobacteriota
OTU1507	3	58	11	2	norank	norank_o__Vicinamibacterales	Acidobacteriota

C: *Claopodium rugulosifolium*, O: *Orthotrichum courtoisii*, P: *Polytrichum formosum*, T: *Taxiphyllum giraldii.*

Table S3. Total number of core fungal sequences in four moss-dominated biocrusts.

OTU ID	Number of sequences				Taxonomy		
	C	O	P	T	Genus	Family	Phylum
OTU1059	258	10	98	2	Alternaria	Pleosporaceae	Ascomycota
OTU193	438	26	25	8	Apiospora	Apiosporaceae	Ascomycota
OTU787	1	26	79	31	Archaeorhizomyces	Archaeorhizomycetaceae	Ascomycota
OTU1370	23	4	9	8	Aspergillus	Aspergillaceae	Ascomycota
OTU1282	17	2	1	1	Chordomyces	Plectosphaerellaceae	Ascomycota
OTU674	2	1180	2561	8	Cladophialophora	Herpotrichiellaceae	Ascomycota
OTU680	47	8486	17531	162	Cladophialophora	Herpotrichiellaceae	Ascomycota
OTU1323	1828	187	1394	104	Cladosporium	Cladosporiaceae	Ascomycota
OTU115	384	110	3	2	Clonostachys	Bionectriaceae	Ascomycota
OTU862	318	3	163	2	Curvularia	Pleosporaceae	Ascomycota
OTU1380	12	121	134	34	Cyphellophora	Cyphellophoraceae	Ascomycota
OTU1477	1	7	3	15	Cyphellophora	Cyphellophoraceae	Ascomycota
OTU1503	9	78	127	92	Cyphellophora	Cyphellophoraceae	Ascomycota
OTU617	5	7	2	6	Cyphellophora	Cyphellophoraceae	Ascomycota
OTU631	100	102	27	3	Cyphellophora	Cyphellophoraceae	Ascomycota
OTU867	7	154	13	2	Cyphellophora	Cyphellophoraceae	Ascomycota
OTU1437	1	24	28	47	Devriesia	Teratosphaeriaceae	Ascomycota
OTU706	1	62	1	7	Devriesia	Teratosphaeriaceae	Ascomycota
OTU800	512	17081	108	15	Dokmaia	Pleosporales_fam_Incertae_sedis	Ascomycota
OTU1024	3093	126	1220	6	Epicoccum	Didymellaceae	Ascomycota
OTU1456	3	20	10	2	Epicoccum	Didymellaceae	Ascomycota
OTU1331	61	204	191	1	Fusarium	Nectriaceae	Ascomycota
OTU649	35	42	12	3	Fusarium	Nectriaceae	Ascomycota
OTU141	28	252	2	2	Fusidium	Xylariales_fam_Incertae_sedis	Ascomycota
OTU967	26	2	15	1	Gibellulopsis	Plectosphaerellaceae	Ascomycota
OTU1251	2	3	6	1	Letendraea	Didymosphaeriaceae	Ascomycota
OTU1230	8	20	286	6	Microsphaeropsis	Pleosporales_fam_Incertae_sedis	Ascomycota
OTU697	246	44	181	303	Neopestalotiopsis	Sporocadaceae	Ascomycota
OTU46	1963	578	95	44	Nigrospora	Trichosphaeriaceae	Ascomycota
OTU824	75	295	29	7	Nigrospora	Trichosphaeriaceae	Ascomycota
OTU355	179	5	602	2	gOchroconis	Sympoventuriaceae	Ascomycota
OTU72	143	275	3	4	Paraconiothyrium	Didymosphaeriaceae	Ascomycota
OTU825	195	41	24	5	Paraconiothyrium	Didymosphaeriaceae	Ascomycota
OTU1219	27	4	2264	9	Paraphaeosphaeria	Didymosphaeriaceae	Ascomycota
OTU288	106	158	7	3	Paraphaeosphaeria	Didymosphaeriaceae	Ascomycota
OTU1212	284	7	164	72	Penicillium	Aspergillaceae	Ascomycota
OTU1413	7	3	3	34	Penicillium	Aspergillaceae	Ascomycota

OTU1472	365	3216	472	200	Penicillium	Aspergillaceae	Ascomycota
OTU1571	53	77	12	136	Penicillium	Aspergillaceae	Ascomycota
OTU763	12	3	1	13	Penicillium	Aspergillaceae	Ascomycota
OTU806	15	8	1	2	Penicillium	Aspergillaceae	Ascomycota
OTU837	28	11	34	2	Penicillium	Aspergillaceae	Ascomycota
OTU971	21	4	1825	94	Penicillium	Aspergillaceae	Ascomycota
OTU1308	3	1	615	1	Periconia	Periconiaceae	Ascomycota
OTU1319	54	74	105	5	Periconia	Periconiaceae	Ascomycota
OTU255	53	2	38	3	Periconia	Periconiaceae	Ascomycota
OTU378	13	13	5	1	Periconia	Periconiaceae	Ascomycota
OTU698	1	5	1	3	Periconia	Periconiaceae	Ascomycota
OTU929	54	62	686	10	Periconia	Periconiaceae	Ascomycota
OTU550	5888	15	153	37	Pestalotiopsis	Sporocadaceae	Ascomycota
OTU990	12	8	54	2	Phaeosphaeriopsis	Phaeosphaeriaceae	Ascomycota
OTU670	16	240	20	1	Plectosphaerella	Plectosphaerellaceae	Ascomycota
OTU1082	178	93	119	2	Pseudopithomyces	Didymosphaeriaceae	Ascomycota
OTU702	298	693	66	3	Pseudopithomyces	Didymosphaeriaceae	Ascomycota
OTU730	17	44	5	37	Pyrenochaetopsis	Cucurbitariaceae	Ascomycota
OTU836	158	9092	29	3	Pyrenochaetopsis	Cucurbitariaceae	Ascomycota
OTU527	15	13	4	9	Roussoella	Thyridariaceae	Ascomycota
OTU583	1	194	26	2	Sarocladium	Hypocreales_fam_Incertae_sedis	Ascomycota
OTU1325	7	31	179	1	Scolecobasidium	Sympoventuriaceae	Ascomycota
OTU1179	32	73	333	83	Setophoma	Phaeosphaeriaceae	Ascomycota
OTU1348	29	5	16	19	Strelitziana	Chaetothyriales_fam_Incertae_sedis	Ascomycota
OTU468	5	4	43	16	Strelitziana	Chaetothyriales_fam_Incertae_sedis	Ascomycota
OTU1470	1097	293	171	3278	Talaromyces	Trichocomaceae	Ascomycota
OTU1593	429	4	76	27	Talaromyces	Trichocomaceae	Ascomycota
OTU243	10	2	2	1	Talaromyces	Trichocomaceae	Ascomycota
OTU771	97	686	17	762	Talaromyces	Trichocomaceae	Ascomycota
OTU345	59	1	16	5	Trichaleurina	Sarcosomataceae	Ascomycota
OTU789	436	2923	258	6	Trichoderma	Hypocreaceae	Ascomycota
OTU1090	8	2	13	8	Trichomerium	Trichomeriaceae	Ascomycota
OTU1518	4	1	3	4	Trichomerium	Trichomeriaceae	Ascomycota
OTU1271	1	2	7	1	unclassified	unclassified_c__Dothideomycetes	Ascomycota
OTU1341	1	136	79	1	unclassified	unclassified_c__Eurotiomycetes	Ascomycota
OTU1583	20	4	61	1	unclassified	unclassified_c__Eurotiomycetes	Ascomycota
OTU1616	8	5	2	1824	unclassified	unclassified_c__Eurotiomycetes	Ascomycota
OTU616	3	16901	22	135	unclassified	unclassified_c__Eurotiomycetes	Ascomycota

OTU405	4	1	1	2	unclassified	unclassified_c__Sordariomycetes	Ascomycota
OTU1167	1	1	1	1	unclassified	Botryosphaeriaceae	Ascomycota
OTU365	346	5	3	10	unclassified	Didymosphaeriaceae	Ascomycota
OTU87	494	37	16	7	unclassified	Montagnulaceae	Ascomycota
OTU368	58	2	5	2	unclassified	Periconiaceae	Ascomycota
OTU1143	153	6	2748	1	unclassified	Phaeosphaeriaceae	Ascomycota
OTU693	25	328	2	2	unclassified	Teichosporaceae	Ascomycota
OTU1057	3	1	54	6	unclassified	Teratosphaeriaceae	Ascomycota
OTU1497	4	6	29	292	unclassified	Teratosphaeriaceae	Ascomycota
OTU1534	1	2	5	27	unclassified	Teratosphaeriaceae	Ascomycota
OTU1535	1	14	26	11	unclassified	Teratosphaeriaceae	Ascomycota
OTU1554	6	19	169	93	unclassified	Teratosphaeriaceae	Ascomycota
OTU625	2	1	1	1	unclassified	Teratosphaeriaceae	Ascomycota
OTU1102	10	1	12	1	unclassified	unclassified_o__Capnodiales	Ascomycota
OTU1350	1	6	10	2	unclassified	unclassified_o__Capnodiales	Ascomycota
OTU382	5	2	2	1	unclassified	unclassified_o__Capnodiales	Ascomycota
OTU1522	1	2	1	944	unclassified	unclassified_o__Chaetothyriales	Ascomycota
OTU1376	22	5	9	15	unclassified	unclassified_o__Hypocreales	Ascomycota
OTU158	50	23	6	14	unclassified	unclassified_o__Pleosporales	Ascomycota
OTU341	12	5	3	2	unclassified	unclassified_o__Pleosporales	Ascomycota
OTU1152	2	96	98	9	unclassified	unclassified_o__Xylariales	Ascomycota
OTU1669	563	19	58	247	unclassified	unclassified_o__Xylariales	Ascomycota
OTU1674	4	3	5	156	unclassified	unclassified_o__Xylariales	Ascomycota
OTU575	7	110	3	140	unclassified	unclassified_p__Ascomycota	Ascomycota
OTU11	11	1	7	5	Whalleya	Xylariaceae	Ascomycota
OTU1324	1	11	56	1	Zymoseptoria	Mycosphaerellaceae	Ascomycota
OTU553	862	2582	164	2	Saitozyma	Trimorphomycetaceae	Basidiomycota
OTU393	446	15	853	222	Hannaella	Bulleribasidiaceae	Basidiomycota
OTU1668	10	3	84	485	Hannaella	Bulleribasidiaceae	Basidiomycota
OTU1147	65	5	413	23	Hannaella	Bulleribasidiaceae	Basidiomycota
OTU1052	16	1	217	3	Erythrobasidium	Erythrobasidiaceae	Basidiomycota
OTU264	160	1	11	5	Rhizopogon	Rhizopogonaceae	Basidiomycota
OTU1663	29	4	44	38	unclassified	unclassified_o__Hymenochaetales	Basidiomycota
OTU991	2	10	68	29	Peniophora	Peniophoraceae	Basidiomycota

OTU329	61	9	1	3	Symmetrospora	Symmetrosporaceae	Basidiomycota
OTU581	31	1	20	13	Ganoderma	Ganodermataceae	Basidiomycota
OTU1165	38	1	22	2	Erythrobasidium	Erythrobasidiaceae	Basidiomycota
OTU1291	26	1	25	1	Hannaella	Bulleribasidiaceae	Basidiomycota
OTU1079	3	9	15	13	Tinctoporellus	Polyporaceae	Basidiomycota
OTU889	1	1	34	2	Schizophyllum	Schizophyllaceae	Basidiomycota
OTU1357	19	2	12	1	Coprinellus	Psathyrellaceae	Basidiomycota
OTU907	4	2	21	3	Psathyrella	Psathyrellaceae	Basidiomycota
OTU104	23	1	1	4	Trechispora	Hydnodontaceae	Basidiomycota
OTU1137	6	3	15	5	Loweporus	Ganodermataceae	Basidiomycota
OTU526	7	1	17	2	Coprinellus	Psathyrellaceae	Basidiomycota
OTU1596	9	2	1	13	unclassified	Thelephoraceae	Basidiomycota
OTU1576	2	1	7	7	Microporus	Polyporaceae	Basidiomycota
OTU1183	1	3	7	5	Nigroporus	Steccherinaceae	Basidiomycota
OTU293	7	1	4	4	unclassified	Thelephoraceae	Basidiomycota
OTU348	7	1	7	1	Gymnopilus	Cortinariaceae	Basidiomycota
OTU479	5	1	8	1	Peniophora	Peniophoraceae	Basidiomycota
OTU1587	5	2	3	3	Coprinopsis	Psathyrellaceae	Basidiomycota
OTU1603	3	1	4	3	Hannaella	Bulleribasidiaceae	Basidiomycota
OTU1689	3	3	3	2	Phlebia	Meruliaceae	Basidiomycota
OTU1262	2	2	4	2	Trechispora	Hydnodontaceae	Basidiomycota

OTU741	4	4	1	1	Pseudomerulius	Tapinellaceae	Basidiomycota
OTU589	2	1	2	3	Odontiopsis	Schizoporaceae	Basidiomycota
OTU1105	1	1	3	1	Ganoderma	Ganodermataceae	Basidiomycota
OTU1293	21	13	4	5	Mortierella	Mortierellaceae	Mortierellomycota
OTU1654	7	5	3	4	Mortierella	Mortierellaceae	Mortierellomycota
OTU235	14	6140	122	5992	unclassified	unclassified	unclassified_k__Fungi
OTU1419	5	20	4	18	unclassified	unclassified	unclassified_k__Fungi
OTU1639	2	2	4	5	unclassified	unclassified	unclassified_k__Fungi
OTU98	1	1	4	4	unclassified	unclassified	unclassified_k__Fungi
OTU817	1	2	3	3	unclassified	unclassified	unclassified_k__Fungi

C: *Claopodium rugulosifolium*, O: *Orthotrichum courtoisii*, P: *Polytrichum formosum*, T: *Taxiphyllum giraldii.*
